# Integration of Omics Approaches Enhances the Impact of Scientific Research in Environmental Applications

**DOI:** 10.3390/ijerph19148758

**Published:** 2022-07-19

**Authors:** Agnieszka Gruszecka-Kosowska, Antonis Ampatzoglou, Margarita Aguilera

**Affiliations:** 1 Department of Environmental Protection, Faculty of Geology, Geophysics, and Environmental Protection, AGH University of Science and Technology, Al. Mickiewicza 30, 30-059 Krakow, Poland; 2 Department of Microbiology, Faculty of Pharmacy, Campus of Cartuja, University of Granada (UGR), 18071 Granada, Spain; ampatzoglou@ms.ugr.es; 3 Institute of Nutrition and Food Technology “José Mataix” (INYTA), Centre of Biomedical Research, University of Granada (UGR-INYTA), 18016 Granada, Spain; 4 IBS—Instituto de Investigación Biosanitaria, 18012 Granada, Spain

## 1. Introduction

Research in the field of environmental sciences currently attracts a lot of attention. As the pollution of the environment is already pushing the earth beyond its homeostasis state, remediation and restoration strategies are needed fast to achieve the United Nations 2030 sustainable development goals. Environmental contamination occurs via many direct and indirect pathways, however, the common denominator appears to be anthropogenic activity. Environmental pollution affects our health, which, according to the World Health Organization (WHO), is defined as not only a lack of disease but rather a holistic state of physical, mental, and social well-being [1]. WHO also estimated that 24% of global human deaths are linked to environment-related issues, i.e., air quality, climate changes and related environmental consequences, water supplies, sanitation and hygiene, food safety, safe use of chemicals, soil quality, plant-microbe ecology, and agricultural practices [2]. Lately, the shifting paradigm definition concerning the term global health has appeared. The term One Health has a much broader meaning describing transdisciplinary cooperation to develop a holistic methodology for simultaneous improvement of human, animal, and environmental health [3,4].

Implementing this One Health approach is challenging, but not only because of the necessity to engage scientists from various cross-cutting areas. Even more difficult to overcome is the tendency of researchers to work in silos of discipline-specific related communities in the fields of science, communication, and management [5]. However, as the strategic goals from the United Nations 2030 Agenda for Sustainable Development [6] are being incorporated, existing frameworks and guidelines promote One Health approaches [7]. Nowadays, one of the most spectacular transcendences of disciplinary boundaries is being observed in life sciences.

With the development of analysis techniques, our knowledge and scientific perspective may grow, evolve, or even change. The breakthrough started with the Human Genome Project [8], which revealed that cooperation among various sciences ranging from biology to medicine and informatics could produce striking results [9]. Since then, the number and impact of scientific projects based on the most advanced research techniques have risen significantly [10]. One of the most spectacular examples of expanding scientific knowledge based on new scientific methods was the discovery of the human gut microbiome and its interactions with vital axes functioning in the human body [11]. The role of the microbiota is now recognised to be far more than what was initially believed; simply “improving” digestion through, for instance, the fermentation of non-digestible substrates such as dietary fibres and endogenous intestinal mucus [12]. The gut microbiota, now a suggested endocrine organ capable of producing and regulating hormones, plays an essential role in food digestion, synthesis of vitamins, pathogen displacement, and influences functions of distant systems and organs [13]. The human microbiome plays a role in many more physiological and clinical states and is even called “the second brain” [14] and “the second liver” [15]. The human microbiome may affect the balance between health and disease among individuals, including the occurrence and severity of illness/metabolic disorders such as obesity [16,17,18,19,20,21], due to the interactions of microbial metabolites and their impact on host physiology [15].

On the other hand, when the cumulative exposure to xenobiotics disrupts the state of homeostasis of the microbiome, dysbiosis might occur, which is associated with disorders and diseases [22,23,24,25,26,27,28,29]. Widely investigated are also the associations between the human microbiome and xenobiotics affecting the host [30,31]. Moreover, environmental exposure and the related interaction with host genetic factors may have an essential role in common chronic diseases [32]. This environmental impact on human health was introduced in 2005 and dubbed exposome, encompassing all environmental exposures (non-genetic factors including lifestyle factors) in the course of life from the prenatal period onwards [33,34]. Later refinements of the definition of exposome focused on cumulative biological responses, the inclusion of behaviour, and endogenous processes [35]. The latest achievements in research technology allowed exposomes to become a novel research paradigm in biomedical sciences [36]. Nevertheless, significant challenges in exposome research relate to the multiple life stages considered, the repeated measurements of biomarkers, the integration of data from biological pathways, and the development of statistical and bioinformatics tools [37].

## 2. Omics Approaches in Environmental Research

The progress and resulting scientific evidence discussed above would not have been possible without omics technologies. Omics technologies have provided high-value data and have allowed for remarkable achievements in life sciences. Today, there is an excellent potential for transferring curated omics approaches to environmental. Therefore, there is an excellent potential for the transfer of know-how and use of omics in environmental research for expanding and gaining key knowledge through innovative scientific and high-throughput methods. In this field, due to the contamination of vast areas with various chemicals and their unintentional mixtures, environmental remediation is not an easy task [38]. One of the critical issues is the cost of remediation action. Moreover, for the successful implementation of remediation plans, a complete understanding of factors affecting the growth, development, or dynamics of microbial communities in polluted areas becomes mandatory [39]. With the development of analysis techniques, including omics approaches, unanswered scientific questions and lack of existing knowledge can be addressed. One of the key issues in environmental remediation is that chemicals may transform and migrate in various compartments of the environment. While scientific knowledge can deal with single chemicals, to a certain extent, unintended mixtures in the environment have been highlighted as a recent challenge [40]. Moreover, the exposome, being the next dimension needed to be incorporated into environmental research, poses additional challenges [41].

**Figure 1:**
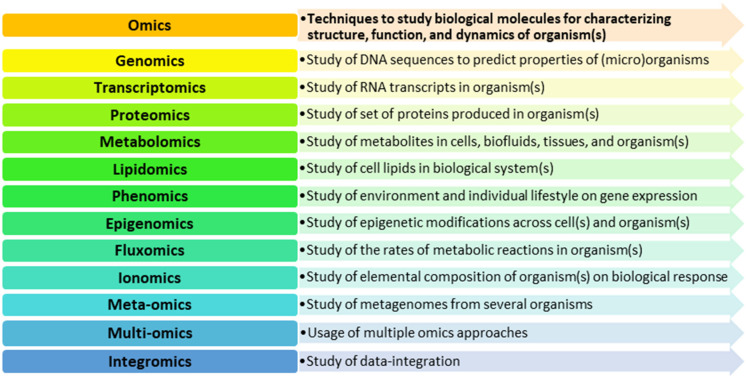
Omics methods with potential application in environmental research.

The remarkable progress in the discipline of life sciences was undoubtedly possible due to the development of so-called omics techniques. The term “omics” refers to several methods used to characterise and describe the roles of different types of molecules in an organism [42]. Based on the current state of knowledge, numerous branches of omics techniques are available for application and integration (Figure 1).

The successful application of omics technologies in life science research and the fact that human, animal, and plant health is inseparably connected to the living environment, which is also the premise of One Health, further highlights the necessity of expanding the use of omics in environmental research. Omics applications in environmental research may effectively support chemical risk assessment by providing the key input in current analytical frameworks such as Adverse Outcome Pathways (AOPs) or Source-To-Outcome (STO) pathways [43,44]. Omics approaches offer a new perspective on microbial communities and could revolutionise the understanding of complex and diverse ecosystems [45], as well as processes such as bioremediation [46]. Analysing biological phenomena through a single omics approach can provide an understanding of biological mechanisms in response to environmental exposure and alterations in ecosystems [47]. Using multi-omics approaches might even lead to a paradigm shift in understanding disease and related exposure factors, e.g., through precision medicine, early and accurate diagnosis biomarkers, and exposure monitoring [47]. Genomics, metagenomics, metabolomics, transcriptomics, proteomics, and multidisciplinary approaches are considered crucial scientific tools for characterising the function, metabolism, and composition of microbiomes in relation to environmental management, monitoring, and repair [48]. The emerging application of these omics methods to environmental research has shown potential in forecasting organism metabolism in contaminated areas and is also considered promising in multiple bioremediation processes [39]. Metabolomics has been used to establish models for predicting microbial activities under bioremediation strategies [49]. For a better understanding of how and why microbes respond to environmental pollutants, advances in fluxomics, genomics, metabolomics, meta-proteomics, meta-transcriptomics, and bioinformatics are required [39]. Fluxomics is also expected to provide results for developing biological systems and systems biology [49]. Metabolomics is crucial for investigating interactions between the genetic background and exogenous and endogenous factors within human health [36]. Through non-targeted metabolomics, a simultaneous overview of both external factors and the associated phenotypic variations is possible due to analysing thousands of exogenous compounds and endogenous metabolites altered by xenobiotic exposure [36]. Omics-based technologies can enormously increase the potential achievements when used in environmental monitoring to capture the biological response of ecosystem perturbations [50].

Undoubtedly, applying new approaches to address a scientific question does not come without challenges. Using omics technologies in research generates a tsunami of scientific data that vastly expands the available information. However, in the quest for new knowledge, some interpretation challenges must be overcome. Due to the vast amount and complexity of data, their analysis requires special techniques based on machine learning and big data [51]. In the case of single omics, data handling must address data filtering and cleaning issues, curation, imputation, transformation, normalisation, and scaling [52]. Besides single omics hurdles, multi-omics analysis has also to overcome the additional challenges of data integration, fusion, clustering, visualisation, and functional characterisation [53]. Thus, it is crucial to work in multidisciplinary teams, where one of the key people is a specialist from the bioinformatics field [54]. Experience in information technology (IT) programming and artificial intelligence (AI) pipeline development for integrative database construction is also necessary [55]. Working with such enormous amounts of data and finding new patterns among observed results requires both adequate tools and critical thinking in interpreting the findings [56,57]. Other challenges include the re-use, storage, analyses, and sharing of such high-dimensional data sets [58] in open access databases within the scientific community, as well as among interdisciplinary research teams.

## 3. Conclusions

Environmental restoration is a matter of necessity, and thus fast and adequate remediation strategies are required. As we are still facing a shortage in scientific knowledge to provide solutions for reducing environmental pollution, single and multi-omics approaches enable researchers to provide novel insights regarding key components of One Health. As omics approaches have made it possible to observe and measure biological systems with unprecedented precision and at continuously decreasing costs, it is only a matter of time before these approaches are fully integrated into environmental sciences. Among the many branches of omics methodologies, genomics, metabolomics, metagenomics, transcriptomics, proteomics, as well as multi-omics and integromics approaches are the most promising for application and progression in this field. The use of omics approaches requires multidisciplinary scientific cooperation, especially bioinformatics, for analysing and interpreting big data obtained through these high throughput techniques. Due to the need to gain new knowledge for mitigation strategies against the consequences of climate change on the environment, the use of omics approaches in environmental science needs to be implemented successfully.
